# New Horned Dinosaurs from Utah Provide Evidence for Intracontinental Dinosaur Endemism

**DOI:** 10.1371/journal.pone.0012292

**Published:** 2010-09-22

**Authors:** Scott D. Sampson, Mark A. Loewen, Andrew A. Farke, Eric M. Roberts, Catherine A. Forster, Joshua A. Smith, Alan L. Titus

**Affiliations:** 1 Utah Museum of Natural History and Department of Geology and Geophysics, University of Utah, Salt Lake City, Utah, United States of America; 2 Raymond M. Alf Museum of Paleontology, Claremont, California, United States of America; 3 School of Earth and Environmental Science, James Cook University, Townsville, Queensland, Australia; 4 Department of Biological Sciences, George Washington University, Washington, D. C. United States of America; 5 Grand Staircase-Escalante National Monument, Bureau of Land Management, Kanab, Utah, United States of America; Paleontological Institute, Russian Federation

## Abstract

**Background:**

During much of the Late Cretaceous, a shallow, epeiric sea divided North America into eastern and western landmasses. The western landmass, known as Laramidia, although diminutive in size, witnessed a major evolutionary radiation of dinosaurs. Other than hadrosaurs (duck-billed dinosaurs), the most common dinosaurs were ceratopsids (large-bodied horned dinosaurs), currently known only from Laramidia and Asia. Remarkably, previous studies have postulated the occurrence of latitudinally arrayed dinosaur “provinces,” or “biomes,” on Laramidia. Yet this hypothesis has been challenged on multiple fronts and has remained poorly tested.

**Methodology/Principal Findings:**

Here we describe two new, co-occurring ceratopsids from the Upper Cretaceous Kaiparowits Formation of Utah that provide the strongest support to date for the dinosaur provincialism hypothesis. Both pertain to the clade of ceratopsids known as Chasmosaurinae, dramatically increasing representation of this group from the southern portion of the Western Interior Basin of North America. *Utahceratops gettyi* gen. et sp. nov.—characterized by short, rounded, laterally projecting supraorbital horncores and an elongate frill with a deep median embayment—is recovered as the sister taxon to *Pentaceratops sternbergii* from the late Campanian of New Mexico. *Kosmoceratops richardsoni* gen. et sp. nov.—characterized by elongate, laterally projecting supraorbital horncores and a short, broad frill adorned with ten well developed hooks—has the most ornate skull of any known dinosaur and is closely allied to *Chasmosaurus irvinensis* from the late Campanian of Alberta.

**Conclusions/Significance:**

Considered in unison, the phylogenetic, stratigraphic, and biogeographic evidence documents distinct, co-occurring chasmosaurine taxa north and south on the diminutive landmass of Laramidia. The famous *Triceratops* and all other, more nested chasmosaurines are postulated as descendants of forms previously restricted to the southern portion of Laramidia. Results further suggest the presence of latitudinally arrayed evolutionary centers of endemism within chasmosaurine ceratopsids during the late Campanian, the first documented occurrence of intracontinental endemism within dinosaurs.

## Introduction

**Figure 1:**
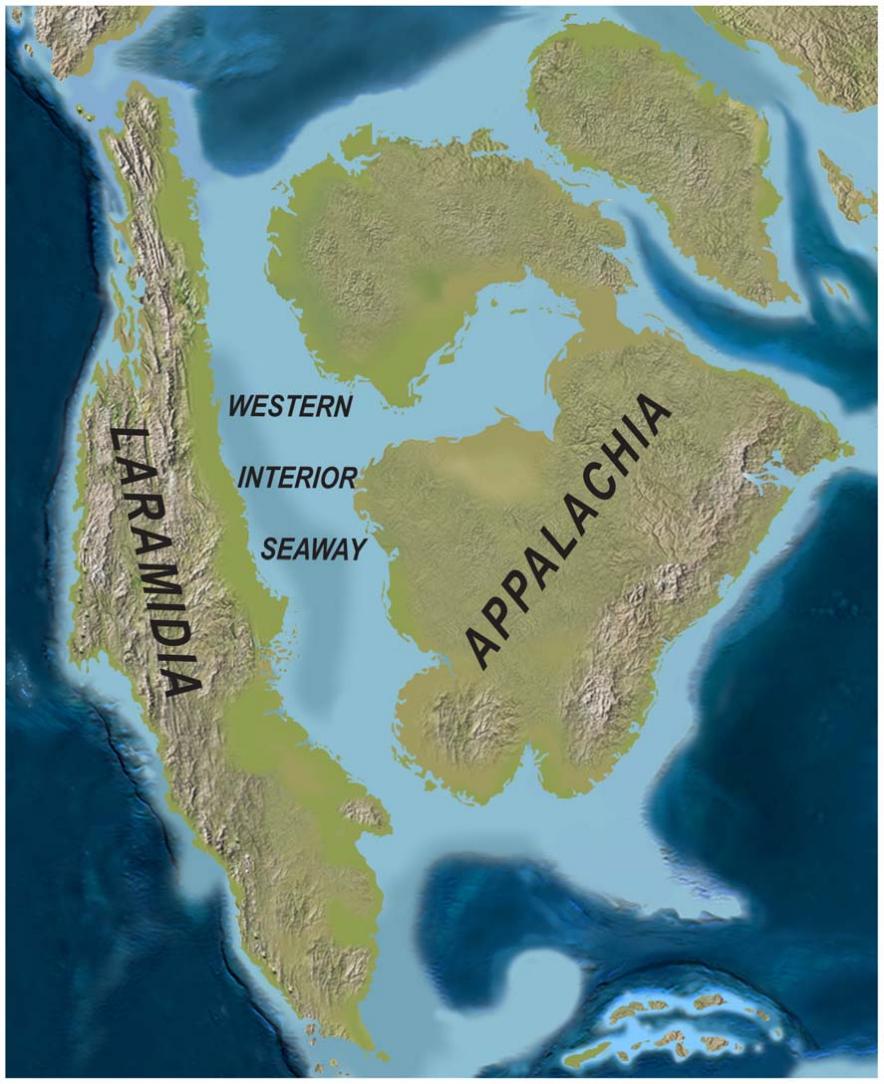
Paleogeography of North America during the late Campanian Stage of the Late Cretaceous (∼75 Ma). Modified after Blakey [1].

For approximately 27 million years of the Late Cretaceous (∼95–68 Ma), elevated global sea levels produced the Cretaceous Western Interior Seaway, a shallow sea that flooded the central portion of North America, forming eastern and western landmasses known as Appalachia and Laramidia, respectively (Fig. 1) [1]. Despite its diminutive size (<20% the present day area of North America) [1], Laramidia was a crucible of evolution, hosting a major evolutionary radiation of dinosaurs that arguably represents the acme of Mesozoic dinosaur diversity. Surprisingly, although many Laramidian dinosaurs were large-bodied (>1000 kg, with many taxa >2000 kg), it has been postulated that Late Cretaceous terrestrial floras and faunas on this landmass were subdivided into distinct northern and southern “biomes,” or “provinces,” with the boundary located approximately in the region of present day northern Utah and Colorado [2]–[4]. Within dinosaurs, the same major clades are present north and south (e.g., hadrosaurids, ceratopsids, ankylosaurids, tyrannosaurids, ornithomimids), but the assemblages appeared largely distinct at the genus and species levels. Almost a half century later, this hypothesis is still challenged [5], [6] and remains poorly tested, in large part because of the dearth of well-dated fossils from southern Laramidia.

Grand Staircase-Escalante National Monument (GSENM) encompasses ∼1.9 million acres of rugged terrain in southern Utah that was the last major region within the contiguous United States to be mapped topographically. Formally designated in 1996, the Monument was established in large part to facilitate preservation and study of its diverse natural resources, both living and fossil. The most fossiliferous terrestrial unit in GSENM is the Upper Cretaceous Kaiparowits Formation, deposited along the eastern margin of Laramidia within 100 km of the seaway [7]. Recent fieldwork has greatly increased the known diversity of fossil vertebrates from this formation, establishing it as one the best known Upper Cretaceous units in the American southwest [8], [9]. Chief among the recent discoveries is a previously unknown dinosaur fauna, including: dromaeosaurid, troodontid, ornithomimid, and tyrannosaurid theropods; hypsilophodont and hadrosaurid ornithopods; ceratopsid and pachycephalosaurid marginocephalians, and ankylosaurian thyreophorans [8]–[12]. Of the 16 dinosaur taxa currently recognized from the Kaiparowits Formation, 10 can presently be identified to genus and species, and all members of this subset represent previously unknown forms.

The evolutionary radiation of ceratopsid dinosaurs was apparently restricted both temporally and geographically; taxa are known predominantly from sediments of latest Cretaceous age (Campanian and Maastrichtian; ∼80–65.5 Ma) in the Western Interior Basin (WIB) of North America, with one exception from the latest Cretaceous of China [13]. Ceratopsids thus appear to have originated and diversified on the “island” continent of Laramidia. With edentulous beaks, hypertrophied narial regions, elongate parietosquamosal frills, and ornamentations on the frill and above the nose and eyes, ceratopsids were among the most specialized and bizarre ornithischian dinosaurs. Two monophyletic clades, Centrosaurinae and Chasmosaurinae, are recognized based on unique suites of morphologic features relating in particular to the elaborate skull roof ornamentations [14]. Here we report the discovery of two “new” genera of chasmosaurine ceratopsids from the Kaiparowits Formation and of Utah, and place these animals into phylogenetic, stratigraphic, and biogeographic context. Based upon robust phylogenetic results presented herein, an additional taxon from the late Campanian of Alberta is also assigned to a new genus.

### Institutional Abbreviations

**NMC**, Canadian Museum of Nature (previously National Museum of Canada), Ottawa, Ontario, Canada; **TMP**, Royal Tyrrell Museum of Palaeontology, Drumheller, Alberta, Canada; **UMNH VP**, Utah Museum of Natural History Vertebrate Paleontology Collections, Salt Lake City, Utah, USA.

## Results

### Systematic Paleontology

**Systematic hierarchy.**

Dinosauria Owen, 1842 [15] *sensu* Padian and May 1993 [16]
Ornithischia Seeley, 1887 [17] *sensu* Sereno 1998 [18]
Ceratopsia Marsh, 1890 [19] *sensu* Dodson, 1997 [20]
Ceratopsidae Marsh, 1888 [21] *sensu* Sereno 1998 [18]
Chasmosaurinae Lambe, 1915 [22] *sensu* Dodson et al., 2004 [14]
*Utahceratops gettyi* gen. et sp. nov.

***Utahceratops gettyi*** **gen et. sp. nov.**

Figures 2, 3, and 4

**Figure 2:**
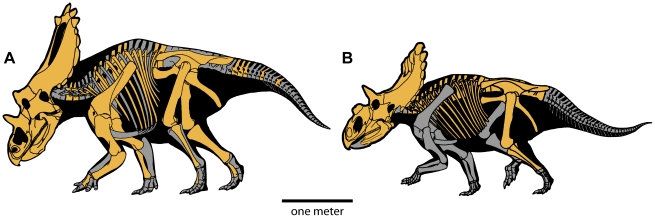
Skeletal elements recovered for *Utahceratops gettyi* n. gen et n. sp. and *Kosmoceratops richardsoni* n. gen et n. sp. *Utahceratops gettyi* is known from six specimens, including two partial skulls, which together preserve about 96% of the skull and 70% of the postcranial skeleton. Highlighted elements are preserved. *Kosmoceratops richardsoni* is known from four specimens, one of which preserves a nearly complete skull and 45% of the postcranium. Scale bar represents one meter.

**Figure 3:**
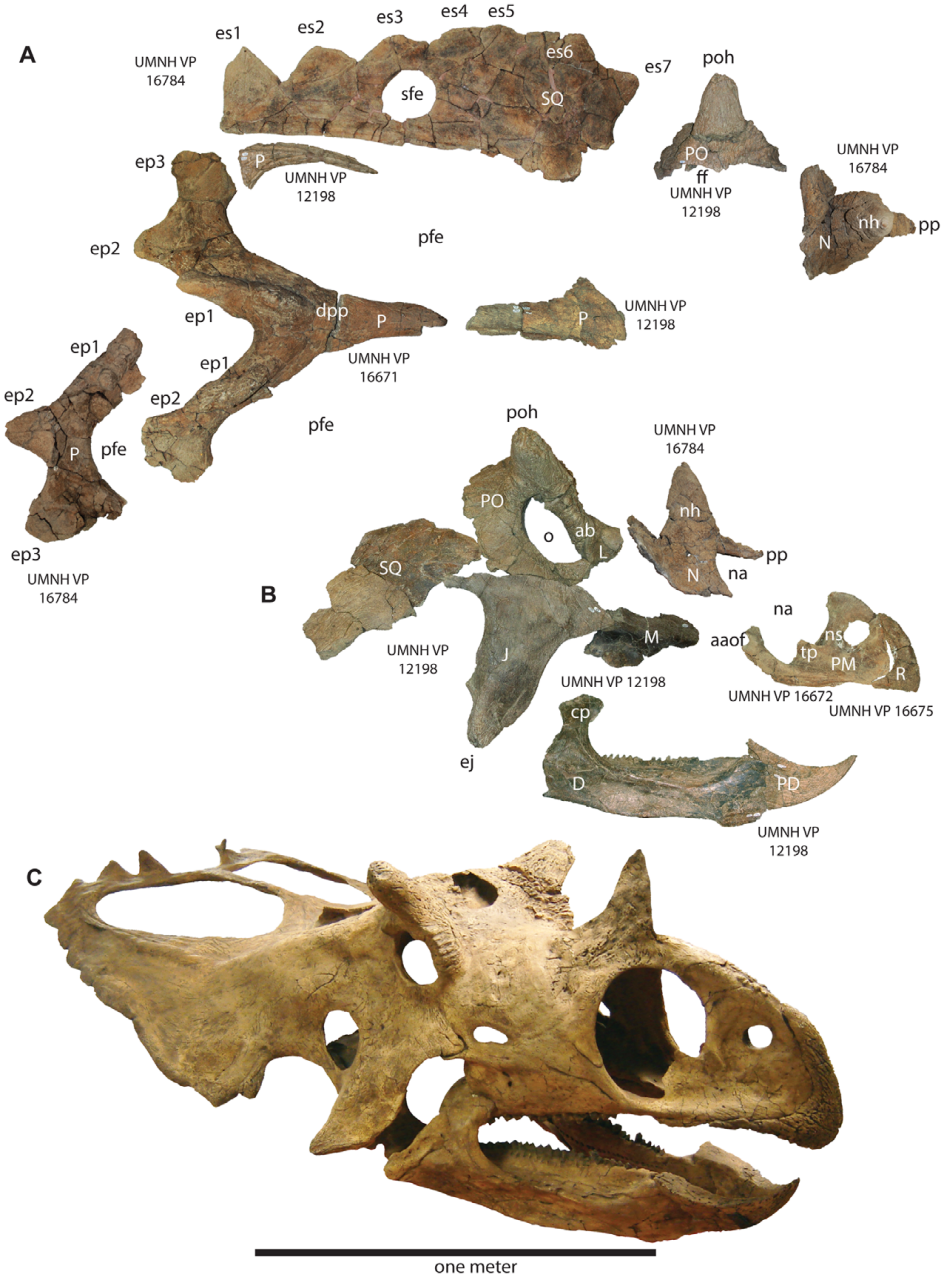
Select craniofacial elements of *Utahceratops gettyi* n. gen et n. sp. **A**. Various cranial elements in dorsal view. **B**. Craniofacial skeleton in lateral view. The orbital region has been photo-reversed for consistency. **C**. Cast of restored skull in oblique view. Scale bar represents one meter. **Abbreviations**: **aaof**, accessory antorbital fossa; **ab**, antorbital buttress; **aof**, antorbital fenestra; **D**, dentary; **dpp**, dorsal parietal process; **cp**, coronoid process; **ej**, epijugal horn; **ep**, epiparietal position 1–3; **es**, episquamosal; **ff**, frontal fontanelle; **J**, jugal; **L**, lacrimal; **ltf**, laterotemporal fenestra; **M**, maxilla; **N**, nasal; **na**, naris; **nh**, nasal horncore; **ns**, narial strut; **o**, orbit; **P**, parietal; **PD**, predentary; **pfe**, parietal fenestra; **PM**, premaxilla; **PO**, postorbital; **poh**, postorbital horncore; **pp**, premaxillary process of nasal; **R**, rostrum; **sfe**, squamosal fenestra; **SQ**, squamosal.

**Figure 4:**
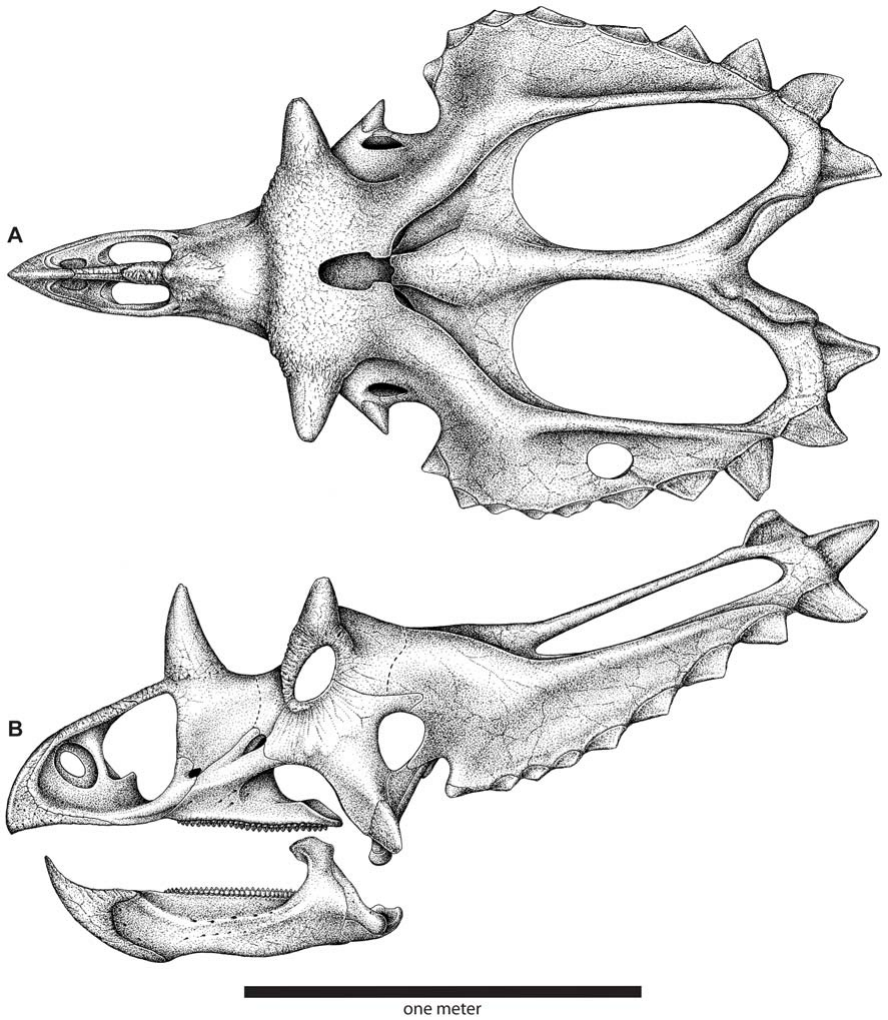
Skull reconstruction of *Utahceratops gettyi* n. gen. et n. sp. In dorsal (**A**) and lateral (**B**) views.

#### Etymology

The generic name refers to Utah, the state of discovery, and *ceratops*, (Greek) meaning “horned face.” The species name honors Mike Getty, who discovered the holotype and who has played a pivotal role in the recovery of fossils from GSENM.

#### Holotype

The holotype specimen is UMNH VP 16784, a partial skull.

#### Type Locality, Horizon and Age

The holotype and assigned specimens occur in the upper portion of the lower unit and the lower portion of the middle unit of the late Campanian Kaiparowits Formation, GSENM, southern Utah, USA.

#### Referred Specimens

UMNH VP 12198, a fragmentary but mostly complete skull about 2.3 m long together with an associated postcranium; UMNH VP 12225, a fragmentary subadult skull including a partial postorbital with the mostly complete supraorbital horncore; UMNH VP 16404, a partial postorbital consisting of the nearly complete supraorbital horncore; UMNH VP 13913, a small, partial juvenile postorbital with complete supraorbital horncore; and several associated elements from bonebed locality 942 consisting of at least two individuals of *Utahceratops*, including a rostrum (UMNH VP 16675), premaxilla (UMNH VP 16672), nasal fragment (UMNH VP 16676), jugal (UMNH VP 16673), squamosal (UNMH VP 16674), and parietal (UMNH VP 16671).

#### Diagnosis

Chasmosaurine ceratopsid diagnosed by the following autapomorphies: nasal horncore caudally positioned, almost entirely behind external naris; supraorbital horncores short, robust, dorsolaterally directed, and oblate in shape with blunt tip; episquamosals on mid portion of lateral frill margin low and extremely elongate (some >10 cm long); and median portion of transverse bar of parietal rostrally curved.

***Kosmoceratops richardsoni*** **gen. et. sp. nov.**

Figures 2, 5, and 6

**Figure 5:**
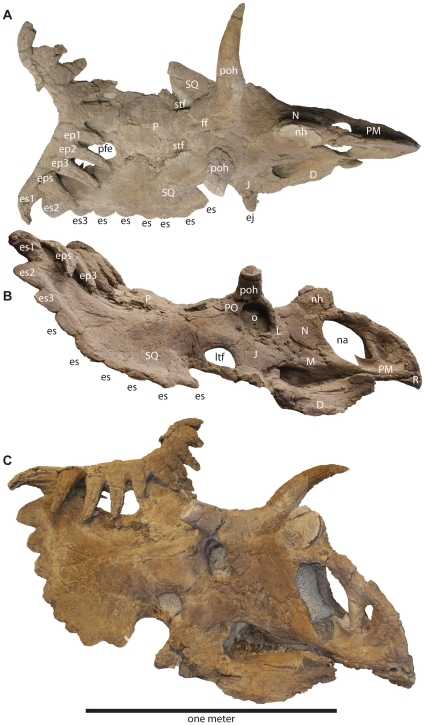
UMNH VP 17000, articulated holotype skull of *Kosmoceratops richardsoni* n. gen et n. sp. In oblique (**A**), dorsal (**B**) and right lateral (**C**) views. Scale bar represents one meter. **Abbreviations**: **D**, dentary; **ep**, epiparietal position 1–3; **eps**, epiparietosquamosal; **es**, episquamosal; **ff**, frontal fontanelle; **J**, jugal; **L**, lacrimal; **ltf**, laterotemporal fenestra; **M**, maxilla; **N**, nasal; **na**, naris; **nh**, nasal horncore; **o**, orbit; **P**, parietal; **pfe**, parietal fenestra; **PM**, premaxilla; **PO**, postorbital; **poh**, postorbital horncore; **R**, rostrum; **sq**, squamosal; **stf**, supratemporal fenestra.

**Figure 6:**
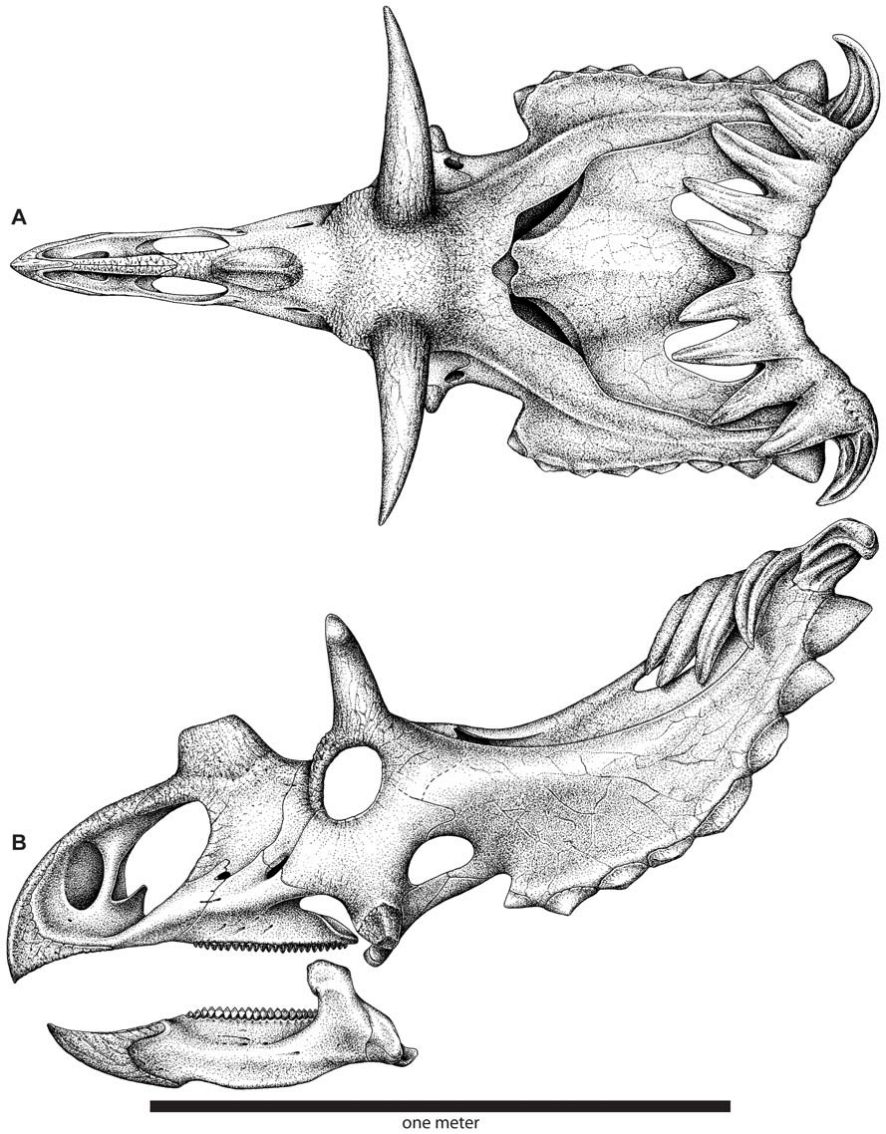
Skull reconstruction of *Kosmoceratops richardsoni* n. gen. et n. sp. In dorsal (**A**) and lateral (**B**) views.

#### Etymology

The generic name refers to *kosmos* (Greek), meaning ornamented, and *ceratops* (Greek), meaning horned face. The specific name honors Scott Richardson, who discovered the holotype and many other significant fossils from GSENM.

#### Holotype

The holotypic specimen is UMNH VP 17000, a nearly complete skull.

#### Type Locality, Horizon and Age

The holotype and assigned specimens occur in the upper portion of the lower unit and lower portion of the middle unit of the late Campanian Kaiparowits Formation, GSENM, southern Utah, USA.

#### Referred Specimens

Referred specimens of *Kosmoceratops richardsoni* consist of: UMNH VP 12198, a disarticulated skull of a subadult individual.

#### Diagnosis

Chasmosaurine ceratopsid diagnosed by the following autapomorphies: internal naris rostrocaudally abbreviated and caudodorsally inclined; nasal horncore transversely constricted, long-based, and blade-like, with flattened distal portion; supraorbital horncores dorsolaterally directed proximally, with a ventral curvature distally tapering to a point; parietosquamosal frill relatively short and broad (maximum width ∼2 times maximum length), with small, caudally positioned parietal fenestrae; parietosquamosal frill with ten well developed processes on caudal margin composed on each side of three procurved epiparietals (ep1-3), one procurved process on the parietosquamosal contact (esp), and one laterally to rostrolaterally directed episquamosal (es1).

***Vagaceratops*** **gen. nov.**

previously *Chasmosaurus irvinensis* Holmes et al., 2001.[23]

#### Etymology

Based upon phylogenetic placement established by the analysis presented herein, a new genus name is indicated (see below). *Vagaceratops* refers to *vagus* (Latin), for wanderer, and *ceratops*, (Greek), meaning “horned face,” in reference to the occurrence of this clade in the north (Alberta) and south (Utah) of Laramidia during the late Campanian. The type species is *Vagaceratops irvinensis* Holmes et al. 2001 [23]. The holotype and assigned specimens occur in the Upper Dinosaur Park Formation, late Campanian, Alberta.

#### Type Species

*Vagaceratops irvinensis* Holmes et al. 2001 [23].

#### Holotype

NMC 41357.

#### Referred Specimens

TMP 87.45.1 and TMP 98.102.8.

#### Locality and Horizon

Upper lithofacies of the Dinosaur Park Formation [23].

#### Revised Diagnosis

Chasmosaurine ceratopsid diagnosed by the following autapomorphies: jugal notch on proximal squamosal broadly rounded and open (not parallel sided); transverse parietal bar straight; epiparietals (ep1-ep3) and epiparietosquamosal (eps) short, forming recurved flat laminae; and predentary length one half that of dentary.

### Description and Comparisons

To date, lower level taxonomic resolution of ceratopsid taxa has been based almost exclusively on craniofacial materials [14]. Thus, the following discussion is limited to key aspects of skull anatomy.

As in most ceratopsids, the circumnarial region of *Utahceratops* includes a relatively large, distally tapering nasal horncore with a pointed terminus. However, the horncore is autapomorphic in being situated almost entirely behind the external naris. The distal half of the horncore possesses a pronounced caudal keel that, combined with the rounded rostral margin, results in a tear-drop shaped cross-section otherwise present only in *Agujaceratops* [24], [25] Ventrolaterally, a thickened region of bone extends ventrally from the horncore onto the remainder of the nasal, extending below the dorsum of the skull almost to the upper margin of the external naris, and the uppermost portion of the external naris shows a distinctive right-angled notch. In contrast, the nasal horncore of *Kosmoceratops* is unique among chasmosaurines in being flat and blade-like, with a transversely narrow, elongate base and rounded distal portion. The internal naris of *Kosmoceratops* differs from that of other ceratopsids in being relatively abbreviated rostrocaudally and distinctly elliptical (instead of subcircular) in overall shape, with a pronounced caudodorsal inclination. The narial strut of the premaxilla is also inclined caudally, a character shared with *Anchiceratops* and *Arrhinoceratops*.

The circumorbital region is highly distinctive for both of the new Utah taxa. Whereas most chasmosaurines possess supraorbital horncores oriented either rostrally or caudally [14], [26], those of *Utahceratops* and *Kosmoceratops* are dorsolaterally directed, superficially similar to the condition in extant *Bison*. However, the two taxa differ greatly in the shape and size of these horncores. Those of *Utahceratops* are relatively short and compressed rostrocaudally, with blunt tips and an overall oblate cross-sectional morphology. Postmortem distortion and pathology can be excluded as explanations for this morphology because four specimens from different localities share the same unique conformation. In contrast, the laterally directed supraorbital horncores of *Kosmoceratops* are considerably more elongate and gracile, curving dorsoventrally and terminating in pointed tips. Immediately rostral and medial to the orbits in both taxa, the skull roof exhibits a pronounced hump, or “forehead,” otherwise present in few ceratopsids (e.g., *Diaboloceratops eatoni*) [27]. As is typical of non-*Chasmosaurus* chasmosaurines [14], both of the Utah taxa possess relatively large epijugal ossifications.

The parietosquamosal frill of *Utahceratops* resembles that of *Pentaceratops* [26] in tapering caudally, with low, elongate episquamosals in the midlateral portion of the frill and a well-developed median embayment on the transverse parietal bar. *Utahceratops* is unique, however, in possessing mid-frill episquamosals with extremely elongate bases (some >10 cm). Additionally, the caudomedian embayment of *Utahceratops* is more pronounced, with a relatively uniform width, in contrast to the tapering condition in *Pentaceratops*. The transverse bar of *Utahceratops* is also unique in being notably curved immediately adjacent to the midline, so as to form a distinct concavity on the rostral surface. The highly derived parietosquamosal frill of *Kosmoceratops* shares several derived features only with *Vagaceratops irvinensis* [23], including a rostrocaudally abbreviated frill with small, caudally placed parietal fenestrae and the presence of 10 well developed hook-like processes (five per side: three on the parietal, ep1-3; one on the squamosal, s1; and another at the boundary of these two elements, esp) on the caudal frill margin, all formed by accessory ossifications. The four medial hooks are directed rostrally, whereas the fifth, lateralmost process (es1) is laterally to rostrolaterally directed. The frill of *Kosmoceratops*, however, shows a more extreme condition than that of *V. irvinensis*, being approximately twice as wide as it is long (as measured on the bone surface), with much smaller, more caudally positioned parietal fenestrae and significantly more elongate and more distinct epi-ossifications on the caudal margin. Chasmosaurines have traditionally been regarded as the “long-frilled” clade within Ceratopsidae. In contrast, *Kosmoceratops* is a chasmosaurine with the shortest frill (relative to total breadth) and smallest parietal fenestrae (relative to total frill area) of any ceratopsid. Moreover, with a total of 15 well developed horns or horn-like structures (1 nasal horncore, 2 postorbital horncores, 2 epijugals, and 10 well-developed epi-ossifications), *Kosmoceratops* possesses the most ornate skull of any known dinosaur.

### Phylogenetics

**Figure 7:**
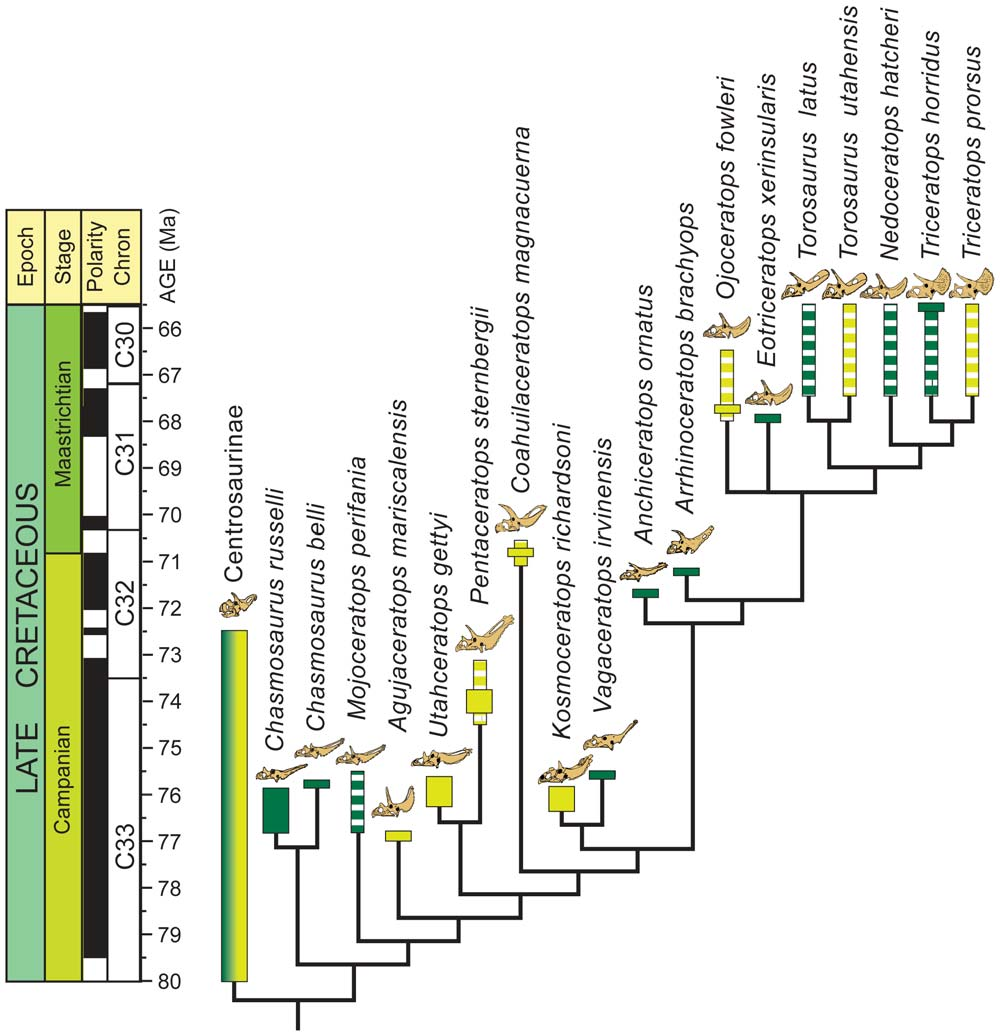
Phylogenetic relationships of *Utahceratops gettyi* n. gen et n. sp., and *Kosmoceratops richardsoni* n. gen. et n. sp. within Ceratopsidae. Strict consensus of 3 most parsimonious trees (tree length  = 263; CI = 0.669; RI = 0.790; RC = 0.529) of an analysis of 148 characters across 7 non-chasmosaurines and 18 chasmosaurines (more than doubling number of characters and taxa relative to any previous analysis of clade). Outgroup taxa and centrosaurines have been collapsed for clarity. Species durations based on first and last documented stratigraphic occurrences correlated where possible to radiometric dates. Solid bars denote species durations known with high degree of confidence. Striped bars denote species durations known with lesser degree of confidence. Solid bars overlaying striped bars indicate that the stratigraphic context of some specimens of the indicated taxon is well established (solid bar) whereas that of others is not (striped bar). Taxa listed in dark green were recovered from the northern portion of the Western Interior; taxa listed in light green are from the southern WIB. See Text S1 for further results of the phylogenetic analysis, including Bremer support and bootstrap values. Stratigraphic data based on Roberts et al. [29] and Sampson and Loewen [12].

A suite of synapomorphies (e.g., premaxilla with narial strut; premaxilla with triangular process; elongate squamosal) clearly place *Utahceratops* and *Kosmoceratops* as members of Chasmosaurinae. A phylogenetic analysis of Chasmosaurinae (Fig. 7; see Materials and Methods below) recovered two species of *Chasmosaurus* (*C. belli* and *C. russelli*) as the basalmost clade, followed by *Mojoceratops* as a distinct branch, and then all remaining chasmosaurines. *Utahceratops* and *Pentaceratops sternbergii* are recovered as sister taxa near the base of the latter clade. *Kosmoceratops* is robustly supported as the sister taxon to *Vagaceratops irvinensis*, and this clade is not closely related to *Chasmosaurus*. The clade of *Kosmoceratops richardsoni* + *Vagaceratops irvinensis* is the sister group to a clade of derived chasmosaurines from the latest Campanian and Maastrichtian, including *Triceratops*. (See also Text S1 and Figure S1.)

## Discussion

The observation of distinct Late Cretaceous dinosaur taxa in the northern and southern regions of the WIB has led to hypotheses of dinosaur provincialism for both the Campanian and Maastrichtian [3], [4], [28]. This idea [3], [4] has been challenged, however, on the basis of both temporal [5], [6] and geographic sampling [6]. With regard to the former, it has been postulated that putative northern and southern dinosaur assemblages during both the Campanian and Maastrichtian were not coeval, but rather reflect a time transgressive taxonomic distribution that has generated the illusion of geographically isolated provinces [5]. Similarly, based upon a statistical analysis of the four most fossiliferous WIB units of Maastrichtian age, it has been argued that the apparently distinct dinosaur assemblages are most likely artifactual, the result of sampling bias between and among geologic formations. We concur that the evidence for latitudinally arrayed dinosaur assemblages during the Maastrichtian is relatively weak, given the poor stratigraphic control and greatly imbalanced sampling. Moreover, retreat of the Cretaceous Western Interior Seaway early in the Maastrichtian resulted in the subaerial reconnection of Laramidia and Appalachia, complicating biogeographic interpretations.

The preceding Campanian stage, in contrast, has yielded an exceptionally diverse assemblage of dinosaur taxa that span a far greater latitudinal range (Alberta to Mexico) and are much better constrained both geographically and stratigraphically [8], [12], [28], [29]. With a dense and relatively well sampled fossil record, the potential for high-precision geochronology, and faunas occupying a peninsular continent, the Campanian WIB represents arguably the best time and place to investigate major questions surrounding the ecology and evolution of Mesozoic terrestrial ecosystems. Until recently, testing of such questions was compromised by a relative dearth of vertebrate remains from the southern portion of Laramidia. The late Campanian-aged Kaiparowits Formation, with abundant exposures in GSENM, has begun to fill this gap.

The Kaiparowits Formation was deposited in the southern region of the Late Cretaceous Western Interior Basin (WIB) at approximately 45° north paleolatitude [29]. Laser-fusion ^40^Ar/^39^Ar ages indicate a late Campanian range for the formation, spanning 76.6–74.5 Ma and corresponding to the Judithian land vertebrate age (Fig. 7) [29]. *Utahceratops gettyi* and *Kosmoceratops richardsoni* occur within a stratigraphic range that spans the upper part of lower unit to the upper part of middle unit of the Kaiparowits Formation, within sediments that date to ∼76.4–75.5 Ma (Fig. 7) [29]. The stratigraphic ranges of these species show considerable overlap, indicating that they were coeval and apparently inhabited the same ecosystems, a rare phenomenon among chasmosaurine ceratopsids from the same formation, and presently unknown within Centrosaurinae [12].

**Figure 8:**
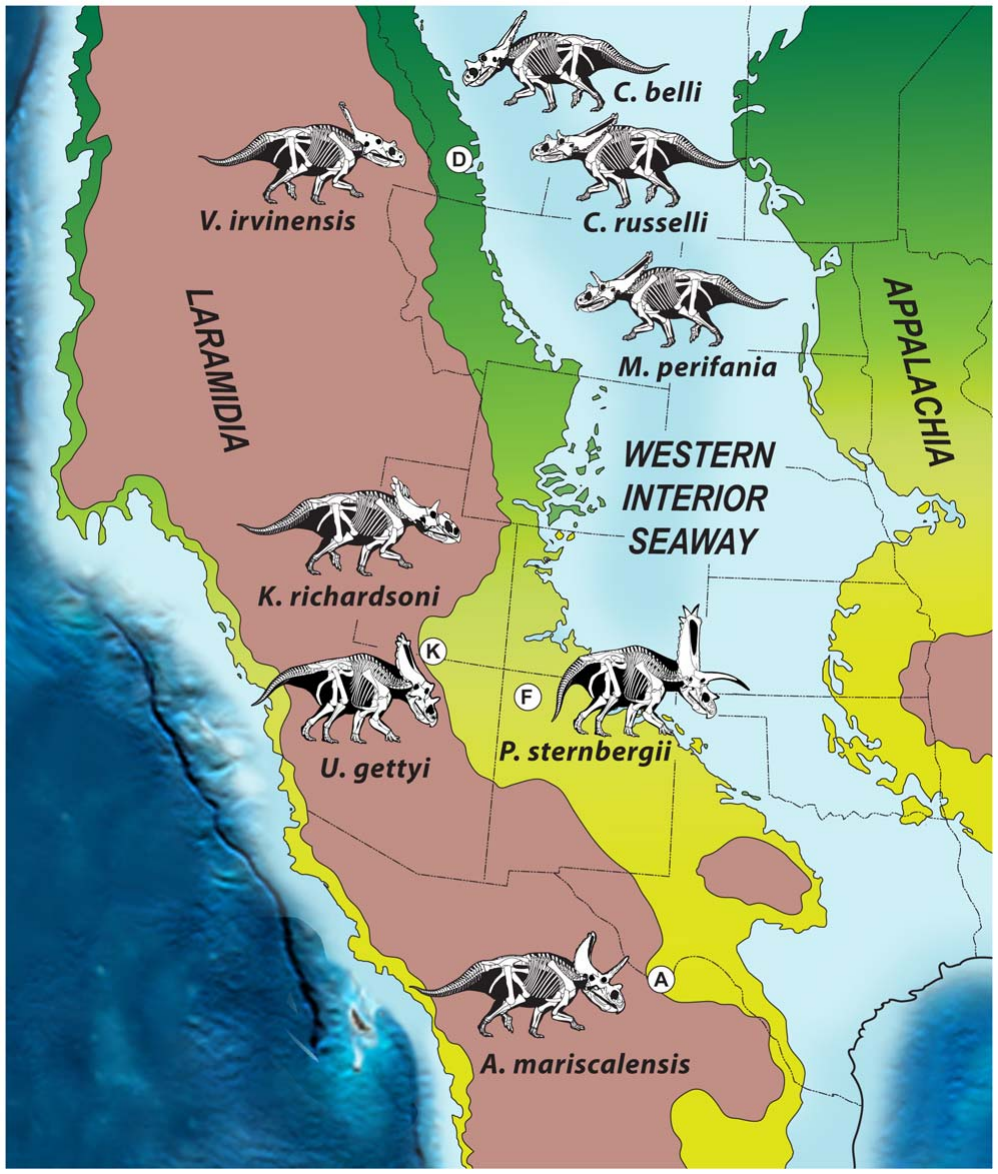
Paleogeography of North America during the Late Cretaceous (∼75 Ma), showing biogeographic distribution of chasmosaurine ceratopsid dinosaurs on the western landmass, Laramidia, during the late Campanian (∼76–73 Ma). Green represents coastal and alluvial plain habitats and reddish brown represents highlands. Present day boundaries of states and provinces are noted, as are the locations of key dinosaur-bearing geologic formations. **Abbreviations**: **A**, Aguja Formation, Texas; **D**, Dinosaur Park Formation, Alberta; **F**, Fruitland-Kirtland Formations; **K**, Kaiparowits Formation, Utah). Modified after Blakey [1].

Geochronologic constraints (i.e., radioisotopic dates and magnetostratigraphy) from other WIB formations demonstrate that the geologically brief interval preserved within the Kaiparowits Formation is contemporaneous with the fossiliferous Dinosaur Park Formation in Alberta, Canada, and penecontemporaneous with other formations to the north (upper lithofacies of Judith River and Two Medicine Formations, Montana), and southeast (Fruitland Formation, New Mexico; upper shale member of the Aguja Formation, Texas) (Figs. 7,8) [30]–[34]. Although current evidence demonstrates that at least some of the vertebrate faunas preserved in these formations experienced significant turnover within the two million year focus interval [35], [36], the geologically brief duration, temporal overlap, and substantial latitudinal span (>20°) permit key comparisons.

*Mojoceratops* and *Chasmosaurus* (*C. russelli* and *C. belli*) are known only from the Dinosaur Park Formation of Alberta [36]–[38]. In contrast, although stratigraphic distributions of the new Utah chasmosaurines overlap with that of *Chasmosaurus russelli* (and possibly with those of *C. belli and Mojoceratops*) (Fig. 7), the Utah taxa are not the closest relatives of each other or of *Chasmosaurus* or *Mojoceratops*. In addition, the stratigraphic range of *Vagaceratops irvinensis* from Alberta overlaps with that of *Pentaceratops sternbergii* from New Mexico, indicating that these taxa were at least partially coeval. This relatively high resolution documentation of coeval, yet distinct species in the northern and southern regions of the WIB constitutes robust evidence refuting the hypothesis of strict time-transgressive occurrences of ceratopsid taxa [5]. Although previous studies have postulated intracontinental endemism in dinosaurs [2]–[4], [28], [39], this is the first documented example based on robust stratigraphic data, demonstrating both geographic disjunction and temporal overlap for distinct taxa from within a single clade. Importantly, of the dozens of species of Campanian dinosaurs described from this landmass, none can currently be placed with confidence in both the northern and southern provinces [9].

Considered in unison, the phylogenetic, stratigraphic, and biogeographic evidence presented here suggests not only dinosaur provincialism (regional faunas) on Laramidia for at least a portion of the late Campanian, but also the presence of northern and southern endemic centers during this interval. With regard to chasmosaurine ceratopsids, whereas species of *Chasmosaurus* occur only in the north, all other basal chasmosaurines are restricted to the southern region of the WIB (Fig. 8). Thus, the phylogenetic evidence implies that all northern taxa from the latest Campanian and Maastrichtian (e.g., *Anchiceratops*, *Torosaurus*, *Triceratops*) evolved from ancestral forms originally restricted to the southern region of the WIB. The combined evidence is most consistent with the following sequence of events pertaining to the origin, dispersal and evolution of Chasmosaurinae: 1) origin on Laramidia 90–80 Ma (the oldest member of the sister clade Centrosaurine dates to about 80 Ma [27]); 2) dispersal of the clade throughout much of Laramidia prior to 77.0 Ma; 3) vicariance due to emplacement of a barrier preventing north-south dispersal by 77.0 Ma; 4) independent evolution of northern and southern chasmosaurines (and presumably other vertebrate clades) within separate latitudinally arrayed endemic centers between at least 77.0 and 75.8 Ma; and 5) dissolution of the barrier approximately 75.7 Ma, followed by a south-to-north dispersal of the *Kosmoceratops* lineage (represented by *Vagaceratops irvinensis*), which ultimately gave rise to all other more derived chasmosaurines.

Among extant vertebrates, large body sizes correlate closely with large individual home ranges and extensive species ranges, likely because of heightened dietary needs [40]–[43]. Yet, despite body sizes that commonly exceeded those of most large-bodied mammals (>1,000 kg), late Campanian dinosaurs on Laramidia apparently possessed relatively diminutive species ranges. This pattern is all the more perplexing when one considers the species diversity of dinosaurs in a typical Laramidian fauna: at least five giant (>2500 kg) herbivores (two ceratopsids, two hadrosaurids, and one ankylosaurid) plus a range of smaller herbivores, together with large- and small-bodied theropod carnivores. Inhabiting a narrow, north-south oriented belt of coastal and alluvial plains, these faunas were subdivided into at least two latitudinally arrayed, semi-isolated regions. Lacking any evidence of a physical barrier to dispersal, and despite the fact that paleo-temperature gradients were markedly reduced relative to those of the present day, dinosaurs appear to have been sensitive to latitudinal zonation of environments [3]. The giant body sizes and undersized species ranges of many Laramidian dinosaurs has important implications for dinosaur biology, suggestive either of low to intermediate physiologies, higher ecosystem primary productivity, or both. The data presented herein suggest further that latitudinal zonation may have persisted for at least 1.5 million years, resulting in distinct endemic centers within the WIB. Additional fossil representatives from the newly discovered Kaiparowits Formation dinosaur fauna (e.g., centrosaurines, hadrosaurids, tyrannosaurids) will be subjected to similar analyses in order to test the hypothesis that other clades exhibit a parallel biogeographic pattern.

The biogeographic pattern supported here—north-south zonation of faunal provinces—on the diminutive landmass of Laramidia requires the presence of a dispersal barrier for nonavian dinosaurs, generally placed approximately at the latitude of northern Utah and Colorado [3], [4]. A recent comprehensive review of vertebrate biogeography on Laramidia during the late Campanian [9] found strong evidence supporting the hypothesis of highly divergent faunas in the northern and southern regions of this landmass, yet the nature of the interface remained unclear. Remaining biogeographic alternatives consist of two or more discrete provinces separated by a zone (or zones) of faunal mixing; or a continuous latitudinal gradient, or cline, with no discrete zones of endemism. To date, no solid evidence exists for a physical barrier to dispersal, although possibilities include: 1) an unidentified, east-west trending mountain range such the Uinta Range of Utah; 2) flooding of the coastal and alluvial plain regions by transgression of the KWIS, temporarily eliminating low elevation terrestrial habitats in the central region of Laramidia; and 3) a major, persistent river system. None of these alternatives appears probable based on available data. Potential evidence of the third alternative occurs in sediments pertaining to the Castlegate Delta just north of the Book Cliffs in Utah [44], although it is difficult to envision a river system acting as a dispersal barrier to terrestrial vertebrates over deep time intervals on the order of 1 million years. More likely is the presence of a paleoclimatic/paleoenvironmental barrier to dispersal, an idea supported by the recovery of divergent pollen types in the north and south of Laramidia [3], [4]. Clearly, the nature of the separation of northern and southern faunal provinces on Laramidia during the late Campanian requires additional investigation that incorporates both paleontological and geological evidence.

## Materials and Methods

### Field Methods and Preparation

The holotype and referred specimens described here were recovered by field crews working with the Utah Museum of Natural History and the Bureau of Land Management. This work was conducted under Bureau of Land Management Assistance Agreements **#** JSA015003 and JSA071004. Many crew members—including Scott Richardson, discoverer of the holotype and referred materials of *Kosmoceratops richardsoni* n. gen. et n. sp.—were volunteers. The specimens were recovered using traditional field methods employed by vertebrate paleontologists, augmented by helicopter airlifts. The blocks were prepared using pneumatic air scribes and needles under magnification.

### Phylogenetic Analysis

In order to assess the cladistic relationships of *Kosmoceratops richardsoni* and *Utahceratops gettyi* relative to other chasmosaurine ceratopsids, a phylogenetic analysis was undertaken. Because numerous features of the skull (e.g., premaxilla and squamosal structure) indicated that the new taxa were members of the clade Chasmosaurinae, selection of ingroup taxa and characters focused on this group. All presently-described chasmosaurine species were included in the analysis, for a total of 18 chasmosaurines. Three centrosaurine ceratopsids—*Albertaceratops nesmoi*, *Centrosaurus apertus*, and *Pachyrhinosaurus lakustai—*were selected as respectively more nested representatives of that clade. Non-ceratopsid members of the analysis included *Leptoceratops gracilis*, *Protoceratops andrewsi*, *Turanoceratops tardabilis*, and *Zuniceratops christopheri*. With the exception of *T. tardabilis*, which was coded from photographs and the literature [45], all other species were coded based on first-hand observations by at least two of the authors. Altogether, over 50 individuals representing 25 species were analyzed (see Text S1 and Figure S1).

A total of 148 equally weighted characters were arrayed across 18 ingroup taxa, more than doubling the number of characters and taxa relative to all previous published analyses of the clade. The characters include both previously identified characters and numerous new characters. 126 of the characters pertain to the skull (including cranium, mandible, and dentition), and the remaining 22 characters relate to the axial and appendicular skeleton. Character 28 was ordered based upon ontogenetic data, and all remaining characters were left unordered.

The character-taxon matrix was assembled in Mesquite v.2.6 [46], and run in the program PAUP* 4b10 for Macintosh PPC [47], with additional analyses conducted in TNT 1.1 [48], [49]. Most parsimonious trees were sought for using the heuristic search command, with *Leptoceratops* constrained as the outgroup. Starting trees were Wagner trees, with a random seed of 1, and 10 replicates. The TBR (tree bisection reconnection) swapping algorithm was used, with 10 trees saved per replication. Bootstrap values were calculated using 10,000 replicates with 10 random addition sequence replicates per bootstrap replicate.

## Supporting Information

Text S1 Extended results of phylogenetic analysis, consisting of: 1) character taxa; and 2) taxon-character matrix.
(0.09 MB DOC)

Figure S1 Phylogenetic relationships of *Utahceratops gettyi* and *Kosmoceratops richardsoni* within Ceratopsidae. Strict consensus of 3 most parsimonious trees (tree length  = 263; CI  = 0.6692; CI excluding uninformative characters  = 0.6602; HI = 0.3308; HI excluding uninformative characters  = 0.3398; RI = 0.7904; RC = 0.5289). Bootstrap values greater than 50% are listed above nodes, and Bremer decay indices greater than 1 are listed below nodes.
(1.96 MB TIF)
