# Supplementary material for: New Horned Dinosaurs from Utah Provide Evidence for Intracontinental Dinosaur Endemism
Source: PLoS One. 2010 Sep 22;5(9):e12292. doi: 10.1371/journal.pone.0012292 (PMC2929175; doi:10.1371/journal.pone.0012292)
Supplement: Text S1 — Extended results of phylogenetic analysis, consisting of: 1) character taxa; and 2) taxon-character matrix. (0.09 MB DOC) [file pone.0012292.s001.doc]

**Supporting Information**

New Horned Dinosaurs from Utah Provide Evidence for Intracontinental Dinosaur Endemism

Scott D. Sampson, Mark A. Loewen, Andrew A. Farke, Eric M. Roberts, Catherine A. Forster, Joshua A. Smith, and Alan L. Titus

**Phylogenetic Analysis: Extended Results**

Three most parsimonious trees were recovered, with a length 263 steps, consistency index of 0.6692, rescaled consistency index of 0.5289, homoplasy index of 0.3308, and a retention index of 0.7904. Character state changes were optimized using accelerated transformation (ACCTRAN), and unambiguous synapomorphies for clades included (character number, with the state at the node indicated after the dot):

Ceratopsoidea: 2.1, 3.1, 32.1, 33.1, 34.1, 35.1, 42.1, 43.1, 44.1, 46.1, 47.1, 52.1, 53.1, 105.1, 106.1, 108.1, 112.1, 117.1, 119.1, 120.1, 125.1, 130.1, 131.1, 144.1

*Turanoceratops* + Ceratopsidae: 123.1

Ceratopsidae: 23.1, 25.1, 26.1, 55.1, 124.1

Centrosaurinae: 4.1, 21.1, 74.1, 79.1, 83.1, 115.1

*Centrosaurus apertus* + *Pachyrhinosaurus lakustae*: 96.1, 102.0

Chasmosaurinae: 6.1, 10.1, 13.1, 17.0, 59.2, 60.1, 64.1, 65.1, 131.2, 143.1

*Chasmosaurus belli* + *Chasmosaurus russelli*: 31.1, 39.0, 100.2, 101.2

*Mojoceratops* + all non-*Chasmosaurus* chasmosaurines: 38.1

All chasmosaurines except *Chasmosaurus* and *Mojoceratops*: 20.1, 47.0, 70.1, 74.1

All chasmosaurines except *Chasmosaurus, Mojoceratops,* and *Agujaceratops*: 14.1, 40.1, 89.2

*Utahceratops* + *Pentaceratops*: 77.1, 88.2, 90.1, 96.1

All chasmosaurines except *Chasmosaurus*, *Mojoceratops, Agujaceratops*, *Utahceratops*, and *Pentaceratops*: 7.1

All chasmosaurines except *Chasmosaurus*, *Mojoceratops, Agujaceratops*, *Utahceratops*, and *Pentaceratops*, and the *Coahuilaceratops*: 20.0, 78.0, 81.0

*Vagaceratops* + *Kosmoceratops*: 61.0, 68.2, 80.1, 94.1, 97.2, 100.2, 101.2, 102.2, 103.1

*Anchiceratops* + *Arrhinoceratops* + *Ojoceratops* + *Torosaurus* + *Nedoceratops* + *Eotriceratops* + *Triceratops*: 8.0, 18.1, 19.0, 21.1, 37.1, 76.1

*Arrhinoceratops* + *Ojoceratops* + *Torosaurus* + *Nedoceratops* + *Eotriceratops* + *Triceratops*: 50.2, 86.0, 89.1, 97.4, 98.0, 99.0, 100.4, 102.4

*Ojoceratops* + *Torosaurus* + *Nedoceratops* + *Eotriceratops* + *Triceratops*: 7.0, 11.1

*Torosaurus* + *Nedoceratops* + *Triceratops*: 60.1, 78.3

*Torosaurus*: 93.1

*Nedoceratops* + *Triceratops*: 69.0

*Triceratops*: 73.0

Only bootstrap values greater than 50 percent are addressed here. The *Kosmoceratops* + *Vagaceratops* clade was recovered 84 percent of the time, the *Chasmosaurus russelli + Chasmosaurus belli* clade was recovered 86 percent of the time, Chasmosaurinae 71 percent, *Pachyrhinosaurus* + *Centrosaurus* 61 percent, Centrosaurinae 73 percent, Ceratopsidae 91 percent, *Turanoceratops* + Ceratopsidae 64 percent, and *Zuniceratops* + *Turanoceratops* + Ceratopsidae 99 percent**.**

**Phylogenetic Analysis: Specimens Included.** Specimens and literature sources used for the scoring of taxa in the phylogenetic analysis. **AMNH**, American Museum of Natural History, New York NY; **ANSP**, Academy of Natural Sciences of Philadelphia, Philadelphia, PA; **CMN**, Canadian Museum of Nature (formerly National Museum of Canada), Ottawa, Ontario, Canada; **CPS**, Colección Paleontológica de Coahuila, at the Museo del Desierto, Saltillo, Coahuila, Mexico; **IGM**, Mongolian Institute of Geology, Ulaan Bataar, Mongolia; **LACM**, Los Angeles County Museum, Los Angeles, CA; **MNA**, Museum of Northern Arizona, Flagstaff, AZ; **MSM**, Mesa Southwest Museum, Mesa, AZ; **NMMNH**, New Mexico Museum of Natural History, Albuquerque, NM; **ROM**, Royal Ontario Museum, Toronto, Ontario Canada; **SMP**, State Museum of Pennsylvania, Harrisburg, PA; **TMM**, Texas Memorial Museum, University of Texas at Austin, Austin, TX; **TMP**, Royal Tyrell Museum of Paleontology, Drumheller, Alberta, Canada; **UALVP**, University of Alberta Laboratory of Vertebrate Paleontology, Edmonton, Alberta, Canada; **UMNH VP**, Utah Museum of Natural History, Salt Lake City, Utah; **USNM**, National Museum of Natural History, Smithsonian Institute, Washington, DC; **UTEP**, University of Texas El Paso, currently at TMM; **YPM**, Peabody Museum, Yale University, New Haven, CT.

| **Taxon** | **Source** |
| --- | --- |

*Leptoceratops gracilis*: CMN 8887, 8889

*Protoceratops andrewsi*: AMNH and IGM various specimens

*Turanoceratops tardabilis*: Sues and Averianov, 2009; Farke et al., 2009

*Zuniceratops christopheri*: MSM bonebed specimens

*Albertaceratops nesmoi*: TMP 2001.26.01

*Centrosaurus apertus*: CMN 348, 8798, 8795; ROM 878; UALVP 11735.

*Pachyrhinosaurus lakustai*: TMP 86.55.258, 87.55.156, 89.55.1234

*Chasmosaurus belli*: CMN 2245, ROM 843

*Chasmosaurus russelli*: CMN 2280, 8800

*Mojoceratops perifania*: AMNH 5401, 5656; TMP 83.25.1

*Agujaceratops mariscalensis*: TMM 43098-1 and UTEP bonebed specimens

*Utahceratops gettyi*: UMNH VP 12198, 12225, 13919, 16671, 16673-75

*Pentaceratops sternbergii*: AMNH 1624,6324; MNA 1747; NMMNH P-27468, 50000

*Coahuillaceratops magnacuerna*: CPS 276, CPS 277

*Vagaceratops irvinensis*: CMN 41357; TMP 87.45.1, 98.102.8

*Kosmoceratops richardsoni*: UMNH VP 12198, UMNH VP 14523

*Vagaceratops irvinensis:* CMN 4135; TMP 87.45.1, TMP 98.102.8

*Anchiceratops ornatus*: AMNH 5251, 5273; CMN 8535; TMP 83.01.01

*Arrhinoceratops brachyops*: ROM 796

*Ojoceratops fowleri*: SMP VP-1575, VP-1828, VP-1719, VP-1828, VP-1865, VP-1875, VP-2090, and other specimens; NMMNH P-4447

*Torosaurus latus*: ANSP 15192; MOR 980, 1122; YPM 1830, 1831

*Torosaurus utahensis*: USNM 15583, 15875, 15887, 16168, 16573-4, 16576

*Eotriceratops xerinsularis*: TMP 2002.57.7

*Nedoceratops hatcheri*: USNM 2412

*Triceratops horridus*: USNM 1201, 1205, 2100; YPM 1820, 1821

*Triceratops prorsus*: LACM 7303, 27428; YPM 1822

**Phylogenetic Analysis: Character List.** Characters used in the phylogenetic analysis are listed below, together with reference to their first use within a cladistic study. For simplicity, original uses within a non-cladistic context (e.g., within a textual description or diagnosis) are not referenced. New characters are indicated in red.

**Dermal Skull Roof**

1. Rostral, extent of dorsal and ventral processes

(0) – triangular in lateral view, with short dorsal and ventral processes

(1) – elongate, with deeply concave caudal margin and hypertrophied dorsal and ventral processes

(Dodson et al., 2004, character 1)

2. Nares, size and position

(0) – small, restricted to dorsal 1/3 of premaxilla, undifferentiated, 10% or less that of basal skull length

(1) – large, expanded to occupy most of the depth of the premaxilla, 15% or greater than basal skull length

(Sereno, 1999, characters 106-108, modified)

3. Premaxillary septum

(0) – absent

(1) – present

(Chinnery and Weishampel, 1998, character 10)

4. Premaxillary septum, shape

(0) – rostrally elongate

(1) – hemicircular

(Dodson et al., 2004, character 4)

5. Premaxillary septum, nasal contribution

(0) – septum formed by premaxilla only

(1) – septum formed by premaxilla and nasal

(new)

6. Premaxilla, narial strut

(0) – absent

(1) – present

(Holmes et al., 2001, character 1)

7. Premaxilla, narial strut orientation

(0) – rostrally inclined

(1) – caudally inclined
(Dodson et al., 2004, character 6)

8. Premaxilla, septal flange

(0) – absent

(1) – present
(Holmes et al., 2001, character 2)

9. Premaxilla, septal flange length

(0) – spans entire caudal margin of narial strut

(1) – restricted to ventral portion of narial strut

(Forster et al., 1993, character 1)

10. Premaxilla, septal fossa

(0) – absent

(1) – present
(Holmes et al., 2001, character 4)

11. Premaxilla, interpremaxillary fossa in premaxillary septum

(0) – absent

(1) – present
(Dodson et al., 2004, character 8)

12. Premaxilla, accessory strut in septal fossa

(0) – no accessory strut

(1) – strut present
(new)

13. Premaxilla, triangular process

(0) – absent

(1) – present
(Forster, 1990, character 21)

14. Premaxilla, triangular process shape

(0) – square

(1) – pinched and triangular with concave facets
(new)

15. Premaxilla, triangular process recess

(0) – absent

(1) – present

(Dodson et al., 2004, character 12)

16. Premaxilla, recess along ventral portion of septum

(0) – absent

(1) – present

(Dodson et al., 2004, character 9)

17. Premaxilla, caudoventral expansion of oral margin

(0) – absent,

(1) – present,

(Forster, 1990, character 6)

18. Premaxilla, position of caudal tip of caudoventral process

(0) – inserts into an embayment in the nasal

(1) – intervenes between nasal and maxilla

(Forster et al., 1993, character 7)

19. Premaxilla, distal end of caudoventral process forked

(0) – absent

(1) – present

(Forster, 1990, character 14)

20. Premaxilla-nasal contact in dorsal view

(0) – premaxillae insert between nasal

(1) – nasals insert between premaxillae

(new)

21. Accessory antorbital fenestra

(0) – present

(1) – absent

(Forster, 1990, character 15)

22. Accessory antorbital fenestra size

(0) – pronounced, penetration of nasal cavity visible in lateral view

(1) – slight penetration, nasal cavity not visible in lateral view

(new)

23. External antorbital fossa, size

(0) – large, 20% or more length of body of maxilla

(1) – greatly reduced or absent, less than 10% length of body of maxilla

(Forster, 1990, character 44)

24. Maxilla, relation of alveolar margin to rostral edentulous margin

(0) – edentulous portion maxilla elevated above level of alveoli

(1) – at same level

(new)

25. Maxilla, diastema on rostral maxilla

(0) – present

(1) – absent

(new)

26. Maxilla, maxillary cavity

(0) – absent

(1) – present

(new)

27. Nasal, ornamentation in adult

(0) – absent

(1) – present

(Forster, 1990, character 26, 27, 28, 126, modified)

28. Nasal, ornamentation type in adult (ORDERED)

(0) – non-pronounced

(1) – distinct horncore

(2) – pachyostotic boss

(Forster, 1990, character 26, 27, 28, 126, modified)

29. Nasal, ornamentation position, measured perpendicular to a horizontal toothrow

(0) – centered dorsal to or caudal to center of endonaris

(1) – centered rostal to center of endonaris

(Forster, 1990, character 29, modified)

30. Nasal, narial spine

(0) – absent

(1) – present

(Forster, 1990, character 22)

31. Facial skeleton, dorsoventral depth in orbital region

(0) – deep, alveolar process of maxilla entirely visible

(1) – shallow, alveolar process of maxilla obscured by jugal

(new)

32. Orbit, orientation

(0) – directed rostrolaterally

(1) – directed laterally
(new)

33. Orbit diameter

(0) – more than 20% of skull length

(1) – less than 15% of skull length

(Makovicky & Norell, 2006, character 3, modified)

34. Lacrimal, size

(0) – large, forms 50% or more of the rostral orbital margin

(1) – small, forms 40% or less of the rostral orbital margin

(Chinnery and Weishampel, 1998, character 1)

35. Postorbital, supraorbital ornamentation in adult

(0) – absent

(1) – present

(Forster, 1990, characters 56 and 57, modified)

36. Postorbital, extent of cornual sinuses in base of supraorbital ornamentation

(0) – sinus space invades frontal and parietal

(1) – sinus space enters postorbital

(Forster, 1990, character 123, modified)

37. Postorbital, position of supraorbital horncore

(0) – centered rostrodorsal or dorsal to orbit, narrow base with caudal margin of supraorbital horncore extending to or only slightly behind caudal margin of orbit

(1) – centered caudodorsal to orbit, broad base with caudal margin of supraorbital horncore extending well behind caudal orbit

(Lehman, 1996, character 9)

38. Postorbital, orientation of supraorbital horncore base

(0) – dorsally directed

(1) – dorsolaterally directed

(new)

39. Postorbital, length of supraorbital horncore

(0) – short, less than 15% basal skull length

(1) – present, elongate, greater than 35% basal skull length

(Forster, 1990, character 58, modified)

40. Postorbital, curvature of supraorbital horncore in lateral view

(0) – caudally recurved

(1) – rostrally curved
(2) – straight

(Forster et al., 1993, character 2, modified)

41. Postorbital, curvature of supraorbital horncore in rostral view

(0) – medially recurved

(1) – laterally curved

(2) – straight

(new)

42. Postorbital, separation from laterotemporal fenestra

(0) – narrowly excluded from fenestra by narrow strip of jugal

(1) – broadly excluded from fenestra by a substantial jugal–squamosal contact (Makovicky & Norell, 2006, character 34, modified)

43. Palpebral, shape

(0) – rod-like, articulates with prefrontal only at its base and projects across dorsal orbit, ligamentous attachment

(1) – blocky, fully fused into dorsal orbital margin, sutural articulation with prefrontal and frontal

(Forster, 1990, character 31, modified)

44. Palpebral, antorbital buttress

(0) – absent

(1) – present

(Sampson, 1995, character 7, modified)

45. Palpebral, extent of antorbital buttress

(0) – present along only anterodorsal portion of orbit

(1) – present along entire anterior portion of orbit

(new)

46. Jugal, size and orientation of jugal body

(0) – projects strongly caudoventrally, does not extend below the level of the maxillary tooth row

(1) – projects nearly ventrally, elongated to extend below the level of the maxillary tooth row

(Makovicky, 2001, character 22)

47. Jugal infratemporal process

(0) – absent

(1) – present, contacts or nearly contacts infratemporal process of squamosal

(Forster, 1990, character 62, modified)

48. Jugal–lacrimal contact

(0) – reduced

(1) – expanded

(Makovicky and Norell, 2006, character 26)

49. Epijugal attachment scar

(0) – large blade like triangle with obtuse angle oriented towards quadratojugal

(1) – scar roughly equilateral in shape

(Sereno 1999, character 113, modified)

50. Epijugal length

(0) – long

(1) – hyperlong

(2) – short

(new)

51. Quadratojugal-squamosal contact

(0) – absent

(1) – present

(new)

52. Laterotemporal fenestra, size and position

(0) – relatively large, diameter 20% or greater that of basal skull length, positioned caudal to orbit

(1) – greatly reduced, diameter 10% or less that of basal skull length, positioned entirely below ventral limit of orbit

(Chinnery and Weishampel, 1998, character 7)

53. Frontal, contribution to orbital margin

(0) – present

(1) – absent

(Forster, 1990, character 51)

54. Frontal, contribution to dorsotemporal fenestra

(0) – present

(1) – absent

(new)

55. Frontal fontanelle leading into supracranial cavity complex

(0) – absent

(1) – present

(Forster et al., 1996, character 3, modified)

56. Frontal fontanelle, shape

(0) – transversely narrow, slit-like

(1) – key-hole shaped, circular or elongate oval

(Forster, 1990, characters 49 and 50, modified)

57. Parietal, anterior extent on dorsum of skull relative to occipital condyle

(0) – rostral end of parietal located well in front of occipital condyle

(1) – rostral end of parietal lies directly over occipital condyle

(new)

58. Squamosal, posterior expansion

(0) – absent or very slight

(1) – present

(new)

59. Squamosal, shape of expanded blade.

(0) – sub-rectangular in outline

(1) – triangular in outline, posteriorly narrowed

(new)

60. Squamosal, length relative to parietal

(0) – squamosal much shorter than parietal, large portion of posterolateral frill made up of parietal

(1) – squamosal slightly shorter than parietal, pmn;y posterolaterl-most margin of frill formed by the parietal

(2) – squamosal and parietal equal in length

(Holmes et al., 2001, character 19, modified)

61. Squamosal forms part of posterior margin of frill

(0) – present

(1) – absent

(new)

62. Squamosal, rostromedial lamina forming the caudolateral floor of dorsotemporal fossa

(0) – absent

(1) – present

(Penkalski and Dodson, 1999, character 9)

63. Squamosal-quadrate contact

(0) – socket-like cotylus on ventrolateral squamosal for ball-like quadrate head

(1) – elongate groove on medial surface of squamosal to receive lamina of quadrate

(Forster, 1990, character 64, modified)

64. Squamosal, thickened, rounded swelling along medial margin

(0) – absent, lateral surface of squamosal flat to slightly convex

(1) – present, lateral surface of squamosal slightly concave

(Forster, 1990, character 90)

65. Parietosquamosal contact, shape in lateral view (new)

(0) – straight

(1) – curved, medially concave

(Forster, 1990, character 119)

66. Parietal, concave median embayment on caudal margin

1. – absent

(1) – present

(new)

67. Parietal, shape of concave median embayment

(0) – shallow, restricted to center of margin

(2) – shallow, entire transverse bar is a V-shaped embayment

(3) – notch-like, restricted to center of margin

(Forster, 1990, character 83, modified)

68. Parietal, location of caudalmost point of caudal ramus

(0) – on midline

(1) – between midline and lateral-most corner
(2) – at lateral-most corner adjacent to squamosal

(Holmes et al., 2001, character 18, modified)

69. Parietosquamosal frill, length relative to basal skull length

(0) – short, .70 or less

(1) – elongate, .85 or more

(Forster, 1990, characters 80, 81, 82, modified)

70. Parietosquamosal frill, location of maximum transverse width

(0) – caudally, at rear margin of frill

(1) – rostrally, in association with proximal half of frill

(2) – widest part in middle or frill relatively equal in width

(new)

71. Parietal, parietal sulci

(0) – absent

(1) – present

(new)

72. Parietal, overall shape

(0) – nearly straight along midline in lateral view and gently arched from side to side

(1) – "saddle-shaped," dorsally concave in lateral view with upturned caudal margin, and arched strongly from side to side.

(new)

73. Parietal fenestra

(0) – absent

(1) – present

(Forster, 1990, character 84)

74. Parietal, rim on medial margin of dorsotemporal fenestra

(0) – absent

(1) – present, well-defined, laterally projecting rim defines medial margin of fenestra

(Forster, 1990, character 86)

75.Parietal, sharp median crest

(0) – present

(1) – absent

(new)

76. Parietal, rostrocaudal thickness of transverse bar at narrowest point

(0) – narrow and straplike, less than 10% total parietal length

(1) – broad, 20% or more of total parietal length

(Holmes et al., 2001, character 22)

77. Parietal, relative rostrocaudal depth of broad transverse bar

(0) – subequal medial to lateral

(1) – tapering so that the narrowest point occurs medially

(new)

78. Parietal, cross-sectional shape of median bar

(0) – diamond-shaped, tapers laterally

(1) – rectangular or subrectangular, margin facing parietal fenestrae thick and oriented sub-perpendicular to parietal surface

(2) – round to lenticular

(3) – v-shaped, opening ventrally

(Holmes et al., 2001, character 24, modified)

79. Parietal, median bar, transverse width

(0) – narrow and straplike, transverse width less than 10% total parietal length

(1) – relatively wide, transverse width 15% or more of total parietal length

(Holmes et al., 2001, character 23)

80. Parietal fenestra, orientation

(0) – long axis directed transversely

(1) – long axis directed axially

(2) – axial and transverse axes equal

(Forster, 1990, character 129, modified)

81. Parietal fenestra, maximum proximodistal diameter

(0) – 35% or less total parietal length

(1) – 45% or more total parietal length

(Forster, 1990, character 129, modified)

82. Parietosquamosal frill, marginal undulations

(0) – absent

(1) – present

(Forster, 1990, characters 89, 114, modified)

83. Parietosquamosal frill, imbrication of undulations

(0) – absent

(1) – present

(Dodson et al., 2004, character 34)

**Epiossifications**

84. Marginal dermal ossifications on parietal and squamosal

(0) – absent

(1) – present

(Forster, 1990, characters 91 and 92, modified)

85. Episquamosals on midlateral squamosal margin

(0) – small, less than 50 mm long in adults

(1) – large and elongate, greater than 90mm long in adults

(new)

86. Episquamosal, location of largest/longest example

(0) – episquamosals subequal in size

(1) – rostralmost episquamosal by far the largest

(2) – caudalmost episquamosal by far the largest

(new)

87. Episquamosal, midlateral, shape

(0) – crescentic or ellipsoidal

(1) – triangular or elongate

(Dodson et al., 2004, character 45, modified)

88. Episquamosal locus S1 shape

(0) – small and crescentic

(1) – low raised D-shaped process

(2) – well developed larger triangular process

(3) – elongate hook

(new)

89. Episquamosal locus S2 shape

(0) – small and crescentic

(1) – low raised D-shaped process

(2) – well developed larger triangular process

(new)

90. Episquamosal locus S2 size relative to other episquamosals

(0) – subequal

(1) – second only to S1 in size, larger than S3

(new)

91. Marginal ossification crossing squamosal-parietal contact

(0) – absent

(1) – present

(Dodson et al., 2004, character 43)

92. Shape of marginal ossification crossing squamosal-parietal contact

(0) – small and crescentic

(1) – present strongly recurved process or gnarled process

(2) – well developed triangular process sometimes with a small peak

(new)

93. Epiparietals, number per side

(0) – three

(1) – five or more

(Holmes et al., 2001, character 28)

94. Epiparietals, fused to adjacent epiparietal at base

(0) – absent

(1) – present

(Holmes et al., 2001, character 29)

95.Epiparietal P0

(0) – absent

(1) – present

(new)

96. Epiparietal locus DPP1

(0) – absent

(1) – present

(Sampson, 1995, character 14, modified)

97. Epiparietal, locus P1 shape

(0) – low D-shaped process

(1) – elongate flattened process or spike

(2) – strongly recurved triangular or recurved low gnarled triangular process

(3) – well developed triangular process

(4) – elongate low process sometimes with a small peak

(Sampson, 1995, character 15, modified)

98. Epiparietal, locus P1 orientation

(0) – caudally, epiparietal oriented in the plane of the frill

(1) – directed rostrodorsally

(Sampson, 1995, character 15, modified)

99. Epiparietal, locus P1 curvature

(0) – straight

(1) – laterally curved

(2) – medially curved

(3) – dorsally curved

(Sampson, 1995, character 15, modified)

100. Epiparietal, locus P2 shape

(0) – low D-shaped process

(1) – elongate flattened process or spike

(2) – strongly recurved triangular or recurved low gnarled triangular process

(3) – well developed triangular process

(4) – elongate low process sometimes with a small peak

(Sampson, 1995, character 16, modified)

101. Epiparietal, locus P2 curvature

(0) – straight

(1) – medially or laterally curved in the plane of the frill

(2) – recurved onto dorsal surface of frill

(Sampson, 1995, character 16, modified)

102. Epiparietal locus P3 shape

(0) – low raised D-shaped process

(1) – elongate spike

(2) – strongly recurved triangular or recurved low gnarled triangular process

(3) – well developed triangular process

(4) – elongate low process sometimes with a small peak

(new)

103. Epiparietal, locus P3 orientation

(0) – caudally, epiparietal oriented in the plane of the frill

(1) – directed rostrodorsally

(new)

**Braincase**

104. Basioccipital,contribution to occipital condyle

(0) – forms approximately 2/3 of occipital condyle

(1) – forms 1/3 of the occipital condyle

(Forster, 1990, character 71)

105.Supraoccipital, contribution to foramen magnum

(0) – forms dorsal margin of foramen magnum

(1) – eliminated from margin by exoccipital-exoccipital contact on midline

(Forster, 1990, character 63)

106. Supraoccipital, ventrolateral processes

(0) – absent

(1) – present

(Sereno, 1999, character 131)

107. Exoccipital, exits for cranial nerves in exoccipital

(0) – three foramina

(1) – two foramina

(Forster, 1990, character 68)

108. Paroccipital process, dorsoventral distal expansion

(0) – distal process only slightly expanded

(1) – distal process expanded to at least .8 two times the depth at its narrowest point

(Forster, 1990, character 66)

**Palate**

109. Ectopterygoid, contributes to palate and contacts the jugal

(0) – present

(1) – absent

(Forster, 1990, character 32)

110. Secondary palate, relative contribution of maxilla

(0) – maxilla forms at least 45% of the secondary palate

(1) – maxilla contributes only to the posterior portion, forms 30% or less of secondary palate

(new)

111. Palatine, shape and relationship to maxilla

(0) – palatine contacts nearly the entire medial surface of the maxilla, restricting size of choanae, rostrodorsal process embraces posterior end of vomer

(1) – palatine contacts only the posterior one-third of medial surface of maxilla, contact with vomer lost, choanae enlarged

(new)

**Lower Jaw**

112. Lower jaw, level of mandibular articulation

(0) – at or slightly below occlusal surface of tooth row

(1) – depressed well below level of occlusal surface of tooth row

(Forster, 1990, character 74)

113. Predentary, length relative to dentary

(0) – equal to or more than two-thirds of dentary length

(1) - less than two-thirds of dentary length

(Chinnery, 1998, character 19; polarity reversed)

114. Predentary, dentary processes

(0) – ventral processes much longer than abbreviated dorsal processes

(1) – dorsal and ventral processes elongate and subequal in length

(new)

115. Predentary, orientation of triturating surface

(0) – nearly horizontal

(1) – inclined steeply laterally

(Dodson, 2004, character 57)

116. Dentary lateral ridge confluent with cutting surface of predentary.

(0) – present

(1) – absent

(new)

117. Dentary, shape of ventral margin in adults

(0) – strongly convexly bowed

(1) – straight

(Forster, 1990, character 73)

118. Dentary, caudal extent of tooth row

(0) – terminates at the center of the coronoid process

(1) – terminates caudal to the coronoid process

(Chinnery and Weishampel, 1998, character 18)

119. Dentary, shape of coronoid process

(0) – short, with gently convex apex, base of ascending ramus rostrocaudally expanded

(1) – tall, expanded at apex into a rostrally projecting hook, base of ascending ramus rostrocaudally restricted

(Forster, 1990, characters 33 and 72, modified)

120. Dentary, separation of body from ascending ramus of coronoid process

(0) – absent

(1) – present

(Makovicky, 2001, character 59, modified)

121. Splenial, shape

(0) – nearly as deep as the body of the dentary, does not contact articular, angular exposed in medial view

(1) – shallow, contacts articular, covers angular in medial view

(Makovicky, 2001, character 62, modified)

122. Prearticular-dentary contact

(0) – absent

(1) – present

(new)

**Dentition**

123. Tooth, number of roots

(0) – one

(1) – two

(Forster, 1990, character 34)

124.Tooth, number of replacements per alveolus

(0) – one or two replacement teeth

(1) – three or more replacement teeth

(Sereno, 1999, character 137)

125. Tooth magazine, case-like alveolar slots for vertical tooth families formed by spongy bone

(0) – absent

(1) – present

(new)

126. Cheek teeth

(0) – spaced

(1) – closely packed, roots abut

(Forster, 1990, character 37)

**Axial Skeleton**

127. Cervical vertebrae, formation of syncervical

(0) – C1-3 fused or tightly articulated, atlantal hypocentrum present as a ventrally placed, wedge-like bone

(1) – C1-3 firmly fused, atlantal hypocentrum forms a complete ring

(Forster, 1990, character 122)

128. Axis, neural spine shape and orientation

(0) – blade-like and nearly vertical, overhangs only rostralmost portion of C3

(1) – blade-like morphology lost, spine steeply angled to reach caudal margin of C3

(Sereno, 1999, character 141)

129. Atlantal rib

(0) – present

(1) – absent

(new)

130. Dorsal vertebrae, shape of centra

(0) – relatively axially elongate

(1) – axially shortened

(new)

131. Sacrum, longitudinal sulcus on ventral surface

(0) – absent

(1) – present

(Lehman, 1989; Sereno, 1999, character 144)

**Pectoral Girdle and Forelimb**

132. Scapula, relative contribution to glenoid fossa

(0) – scapula and coracoid contribute equally

(1) – scapula contributes well over half of the glenoid

(Sereno, 1999, character 145)

133. Scapula, orientation of scapular spine

(0) – runs obliquely across blade

(1) – runs longitudinally along blade

(new)

134. Olecranon process

(0) – relatively small

(1) – enlarged (>one-third of ulnar length)

(Forster, 1990, character 104, modified)

135. Clavicle

(0) – present

(1) – absent

(Sereno, 1999, character 147)

136. Manual and pedal unguals, shape

(0) – taper to distal tip

(1) – dorsoventrally flattened with blunt and rounded distal tips

(Chinnery and Weishampel, 1998, character 64)

137. Manal and pedal penultimate phalanges, shape

(0) – length exceeds width

(1) – width exceeds length

(new)

**Pelvic Girdle and Hind Limb**

138. Ilium, lateral eversion of dorsal margin

(0) – absent

(1) – present

(Forster, 1990, characters 108-109, modified)

139. Ilium, relative lengths of pubic and ischial peduncles

(0) – pubic and ischial peduncles long, extend well below body of ilium approximately the same distance

(1) – ischial peduncle reduced along ventral aspect, pubic peduncle projects further ventrally than ischial peduncle

(new)

140. Pubis, prepubic process

(0) – short and unexpanded distally

(1) – elongate, distal end greatly expanded dorsoventrally

(Forster, 1990, character 111)

141. Pubis, position and length of postpubic rod

(0) – relatively short but extends past ischial peduncle of ilium, arises ventral to acetabulum and lies along ventral and ventromedial margin of ischium

(1) – very abbreviated, terminates at level of ischial peduncle, arises medial to acetabulum and passes entirely medial to ischium

(Forster, 1990, character 110)

142. Pubis and ischium, morphology of contributions to acetabulum

(0) – pubic acetabular surface faces caudolaterally, pubis and pubic process of ischium contribute equally to ventral margin of acetabulum

(1) – pubic acetabular surface faces laterally and forms a partial medial wall to the acetabulum, pubic process of ischium elongate and meets pubis close to anterior margin of acetabulum, ventral portion of pubic acetabular surface lies medial to pubic ramus of ischium

(new)

143. Ischium, cross-sectional shape of shaft

(0) – thick and ovoid

(1) – laterally compressed and bladelike, tapered dorsally

(Forster, 1990, character 112)

144.Ischium, orientation of shaft

(0) – nearly straight or slightly decurved

(1) – broadly and continuously curved

(Forster, 1990, character 113)

145. Femur, morphology of greater and lesser trochanters

(0) – trochanters distinct and located below the level of the femoral head

(1) – trochanters coalesced and level with the femoral head

(Dodson et al., 2004, character 72)

146. Femur, size of fourth trochanter

(0) – large and pendant

(1) – small, reduced to low prominence

(Sereno, 1999, character 154)

147. Femur-tibia proportion

(0) – tibia longer than femur

(1) – femur longer than tibia

(Forster, 1990, character 103)

148. Pes, metatarsal proportions

(0) – length of MT I two-thirds the length of MT II

(1) – MT I reduced to one half or less the length of MT II

(new)

**Phylogenetic Analysis: Taxon Scores**

*Leptoceratops gracilis*

100??0???0??0??010001?000?0??000000??????000?0000000000?00??000000?00000000??????000????????????????????00?0000000000000000000?000000000000?0?000000

*Protoceratops andrewsi*

000??0???0??0??01000010000100000000??????000?0000000000?0110110000?00000100000010000???????????????????000000000000?00000000000000000000000000000000

*Zuniceratops christopheri*

111000???0??0??0?010000?000??0?1111000102111011?0??11?0???????0????????01?01000????0????????????????????11?1???1?0011011??001????1100?????????01?0??

*Turanoceratops tardabilis*

??????????????????????0000??????1?1?00101?1?????????1?0??????????????????????????1????????????????????????????????0???????1011??????????????????????

*Albertaceratops nesmoi*

?111?0???0??0??????01?1011100??11?1?00112111111112111???1110111001011110111100121111000???0010001013030??1?1???1?11011?1???111??????????????????????

*Centrosaurus apertus*

011110???0??0??111001?111111010111100002111111111211111011101110010110101111001211110000000010011120000111111111111011111111111111110111111111011111

*Pachyrhinosaurus lakustai*

011110???0??0??111001?111112010111111????110?1?112?111101110111001011010111100121111??0???0?1001102110010111111?1110111111111111?1?10??11111110111??

*Chasmosaurus belli*

1110010101001000001001111111001111110000211111111211111111211011111210101010010211010111100?00002132230?11111111110111111111111111210?11111111111111

*Chasmosaurus russelli*

1110010101001000001001111111001111110000211111111211111111211011112110101010010211010111100?00002132230?11111??1110111111111111111210?1111111111111?

*Mojoceratops perifania*

1110010101001000001000011?110001111101100111111110111111112110?1112110001010020211010111100?00003103030???????????????????1?????????????????????????

*Vagaceratops irvinensis*

1110011101001?0000100111111100011101?????111111112111?111122001110?211?01210000101010011201101002132221?1111???11101111111?1111111?10?1111???11??11?

*Kosmoceratops richardsoni*

1110011111001100001001111111000111110111211111011111111111220010111211101110001101(01)10213211101002132221????????11?011?111?1?11????????1?????????????

*Agujaceratops mariscalensis*

111001010100100000111?11111100011111011001111101111111??112?1011112?11??1110?20211010?111?0???0????????111111???110?1111??1?111111210????111?11111??

*Utahceratops gettyi*

11100101110?110000110?1111110001111111022111110111?1111111211011112111101110120211011212210?00013133030???1111?111011111111111???1?10??????1??11111?

*Pentaceratops sternbergii*

1110010111011100001101111111000111110011211111011111111111211011112211101110120211011212210?00013133030?11111111110111111111111111210?1111111111?11?

*Coahuilaceratops* *magnacuerna*

11100?1??10????00??1??11111110??1?11??11?1???????????????12??01?1??????01?1??2021101??1?????????????????????????1?0111111???11?????10???????????????

*Anchiceratops ornatus*

11100110?100110001001?11111100011111111111111101111111111122101110?11110121100120101121220120000313303011111111?????????????111111?10?1111?1??111?11

*Arrhinoceratops brachyops*

1110011??1001?0001?01?111?110001111?111111111101121111011122?01110?111101211001201011011100?0000400404011111111?????????????11??????????????????????

*Ojoceratops fowleri*

11??0??????????????0??????111??1??????????111????????????1220?101?????????1100???101101110??00104004040????????111011?111???11??????0???????????????

*Torosaurus latus*

1110010??111???0010?1?111111100111111(01)112111110112111?(01)11121101110?(01)12101011031301011011100?10004004040111111111?1????????1111???1?10???????????????

*Torosaurus utahensis*

?????????????????????????????????11?10112111110112?1??1??12?101110?012101011031?01011011111210104004040????????111?11111??1?111?11??????????????????

*Eotriceratops xerinsularis*

11100100?11011000??0??111111??011?111?1121?111?112???????12?1???1????????????????1011?1???12????????????????????????????????1111????????????????????

*Nedoceratops hatcheri*

11100100?11111100?0?1?111?1110011?1?10112?11110?12?1?11111211?101???021?1111031201011011101???????????????111????????????????1??????????????????????

*Triceratops horridus*

11100100?111111001001?11111110011111101121111101121111111121101010?00211001??????1011011101200104004040111111111110111111111111111211111111111111111

*Triceratops prorsus*

11100100?111111001001?11111110011111101121111101121111011121101010?00211001??????1011011101200104004040111111111110111111111111111??1??????????????1
