# Supplementary figures and images for: New Horned Dinosaurs from Utah Provide Evidence for Intracontinental Dinosaur Endemism
Source: PLoS One. 2010 Sep 22;5(9):e12292. doi: 10.1371/journal.pone.0012292 (PMC2929175; doi:10.1371/journal.pone.0012292)

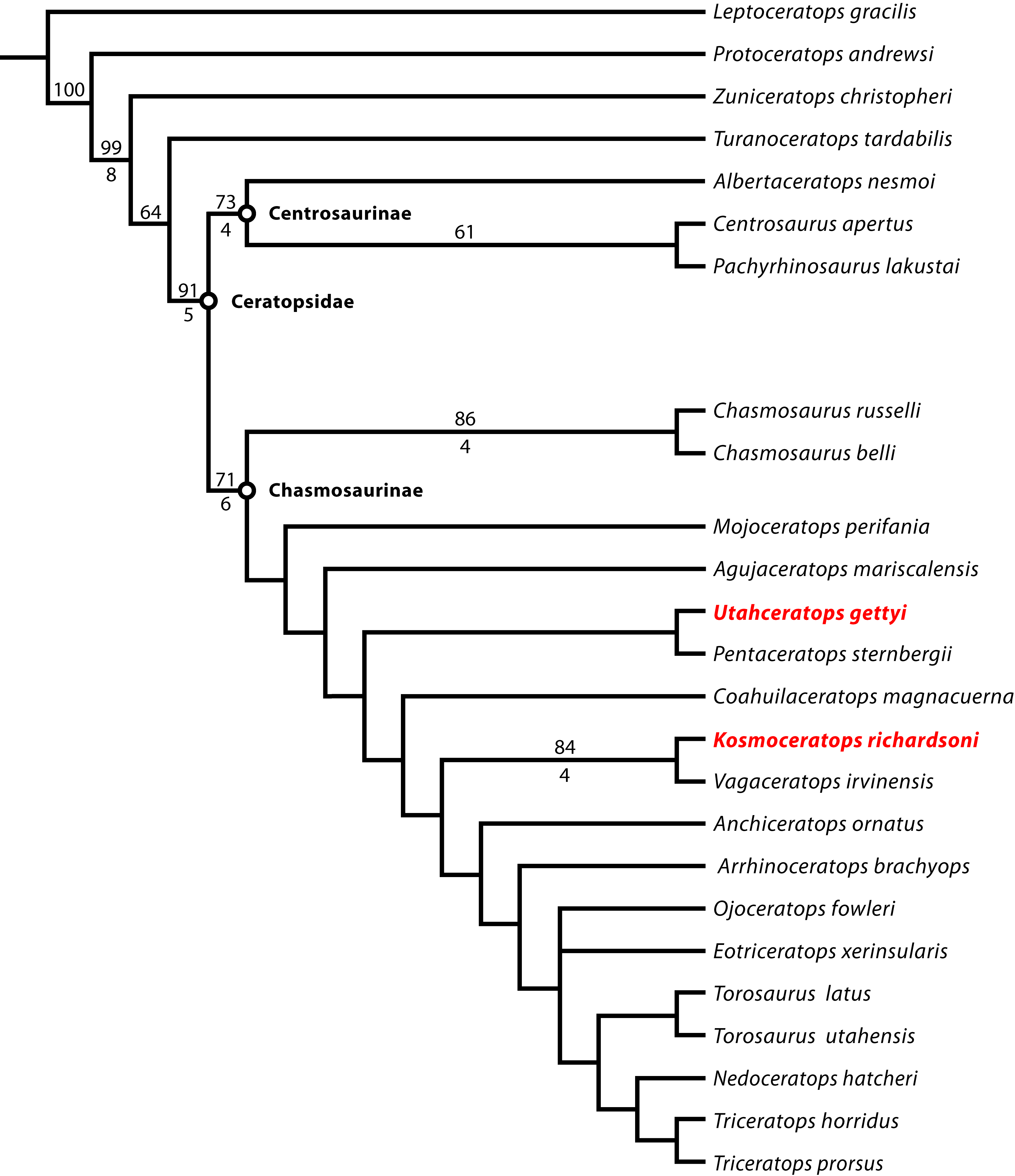

Supplement: Figure S1 — Phylogenetic relationships of Utahceratops gettyi and Kosmoceratops richardsoni within Ceratopsidae. Strict consensus of 3 most parsimonious trees (tree length = 263; CI = 0.6692; CI excluding uninformative characters = 0.6602; HI = 0.3308; HI excluding uninformative characters = 0.3398; RI = 0.7904; RC = 0.5289). Bootstrap values greater than 50% are listed above nodes, and Bremer decay indices greater than 1 are listed below nodes. (1.96 MB TIF) [file pone.0012292.s002.tif]
